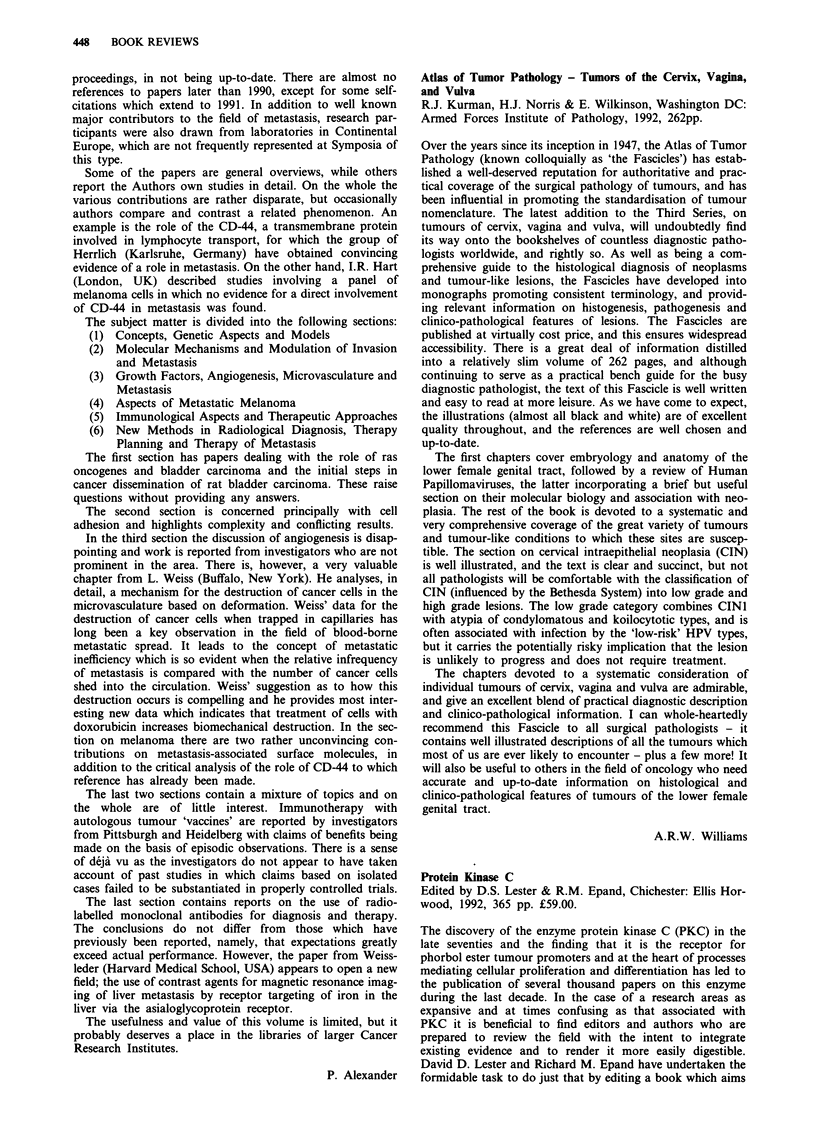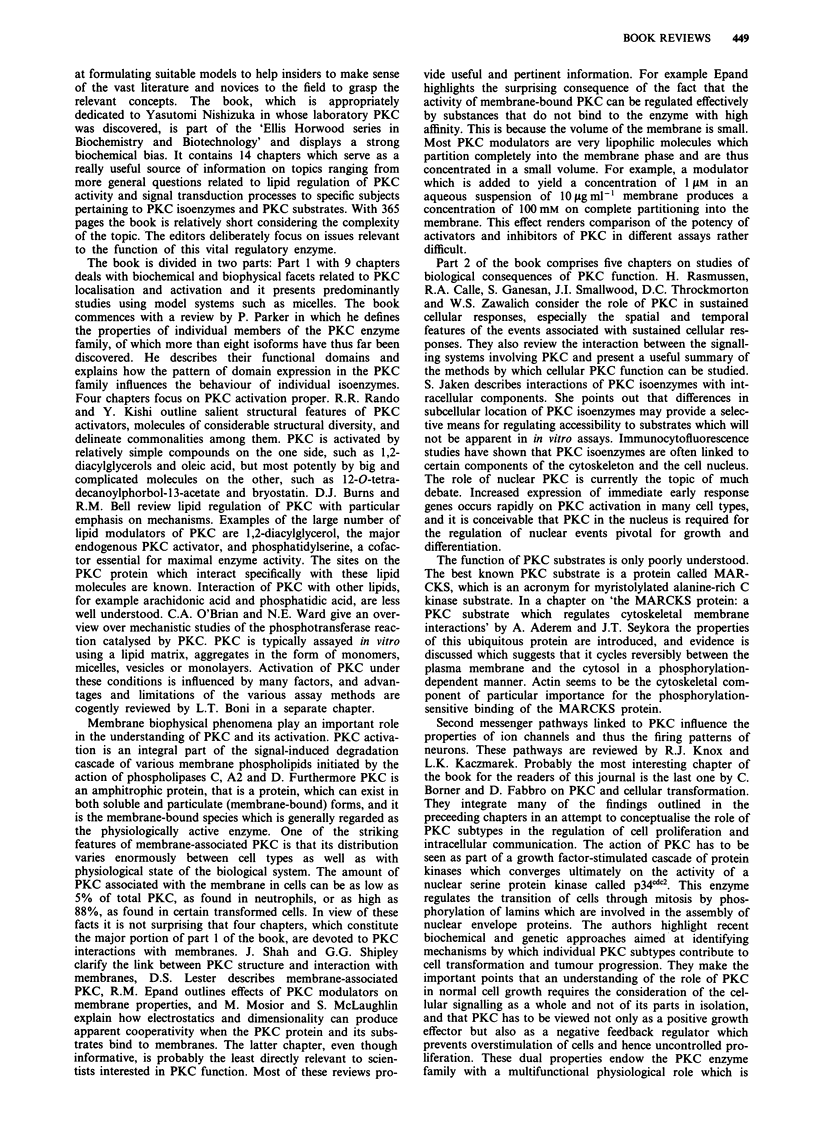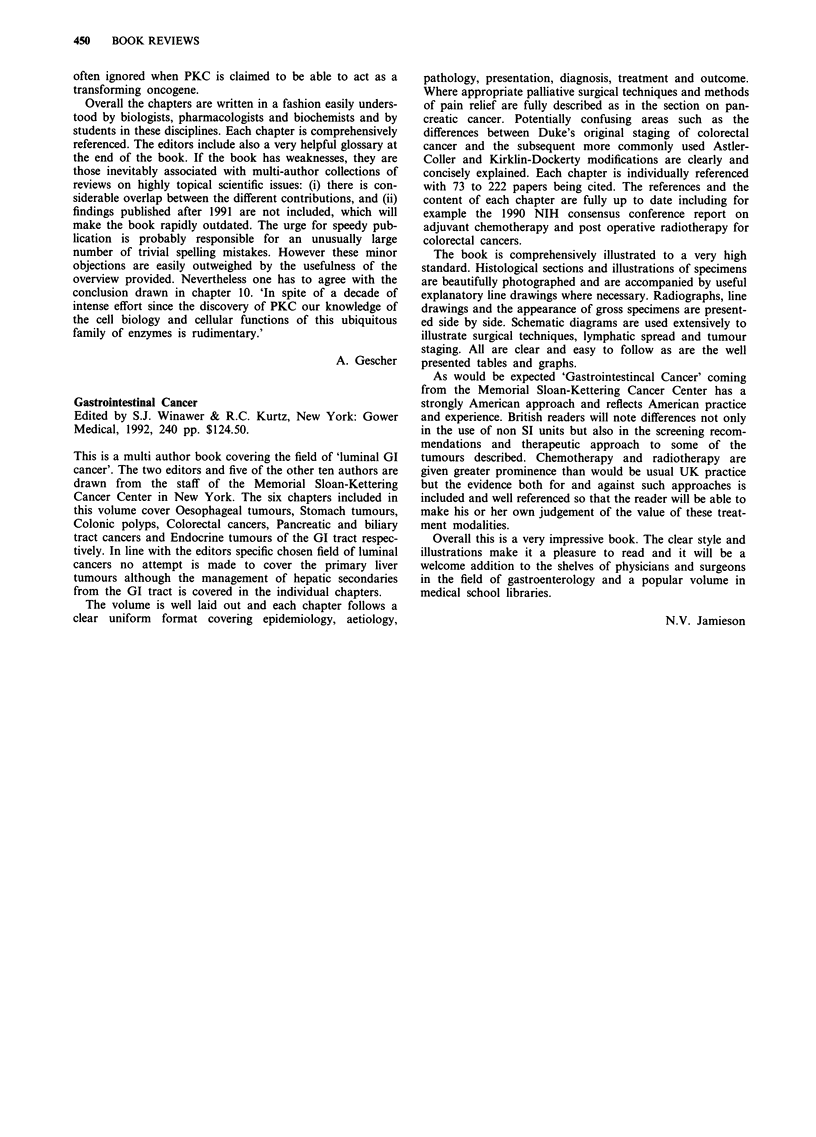# Protein Kinase C

**Published:** 1993-08

**Authors:** A. Gescher


**Protein Kinase C**

Edited by D.S. Lester & R.M. Epand, Chichester: Ellis Horwood, 1992, 365 pp. £59.00.

The discovery of the enzyme protein kinase C (PKC) in the late seventies and the finding that it is the receptor for phorbol ester tumour promoters and at the heart of processes mediating cellular proliferation and differentiation has led to the publication of several thousand papers on this enzyme during the last decade. In the case of a research areas as expansive and at times confusing as that associated with PKC it is beneficial to find editors and authors who are prepared to review the field with the intent to integrate existing evidence and to render it more easily digestible. David D. Lester and Richard M. Epand have undertaken the formidable task to do just that by editing a book which aims at formulating suitable models to help insiders to make sense of the vast literature and novices to the field to grasp the relevant concepts. The book, which is appropriately dedicated to Yasutomi Nishizuka in whose laboratory PKC was discovered, is part of the 'Ellis Horwood series in Biochemistry and Biotechnology' and displays a strong biochemical bias. It contains 14 chapters which serve as a really useful source of information on topics ranging from more general questions related to lipid regulation of PKC activity and signal transduction processes to specific subjects pertaining to PKC isoenzymes and PKC substrates. With 365 pages the book is relatively short considering the complexity of the topic. The editors deliberately focus on issues relevant to the function of this vital regulatory enzyme.

The book is divided in two parts: Part 1 with 9 chapters deals with biochemical and biophysical facets related to PKC localisation and activation and it presents predominantly studies using model systems such as micelles. The book commences with a review by P. Parker in which he defines the properties of individual members of the PKC enzyme family, of which more than eight isoforms have thus far been discovered. He describes their functional domains and explains how the pattern of domain expression in the PKC family influences the behaviour of individual isoenzymes. Four chapters focus on PKC activation proper. R.R. Rando and Y. Kishi outline salient structural features of PKC activators, molecules of considerable structural diversity, and delineate commonalities among them. PKC is activated by relatively simple compounds on the one side, such as 1,2-diacylglycerols and oleic acid, but most potently by big and complicated molecules on the other, such as 12-*O*-tetradecanoylphorbol-13-acetate and bryostatin. D.J. Burns and R.M. Bell review lipid regulation of PKC with particular emphasis on mechanisms. Examples of the large number of lipid modulators of PKC are 1,2-diacylglycerol, the major endogenous PKC activator, and phosphatidylserine, a cofactor essential for maximal enzyme activity. The sites on the PKC protein which interact specifically with these lipid molecules are known. Interaction of PKC with other lipids, for example arachidonic acid and phosphatidic acid, are less well understood. C.A. O'Brian and N.E. Ward give an overview over mechanistic studies of the phosphotransferase reaction catalysed by PKC. PKC is typically assayed *in vitro* using a lipid matrix, aggregates in the form of monomers, micelles, vesicles or monolayers. Activation of PKC under these conditions is influenced by many factors, and advantages and limitations of the various assay methods are cogently reviewed by L.T. Boni in a separate chapter.

Membrane biophysical phenomena play an important role in the understanding of PKC and its activation. PKC activation is an integral part of the signal-induced degradation cascade of various membrane phospholipids initiated by the action of phospholipases C, A2 and D. Furthermore PKC is an amphitrophic protein, that is a protein, which can exist in both soluble and particulate (membrane-bound) forms, and it is the membrane-bound species which is generally regarded as the physiologically active enzyme. One of the striking features of membrane-associated PKC is that its distribution varies enormously between cell types as well as with physiological state of the biological system. The amount of PKC associated with the membrane in cells can be as low as 5% of total PKC, as found in neutrophils, or as high as 88%, as found in certain transformed cells. In view of these facts it is not surprising that four chapters, which constitute the major portion of part 1 of the book, are devoted to PKC interactions with membranes. J. Shah and G.G. Shipley clarify the link between PKC structure and interaction with membranes, D.S. Lester describes membrane-associated PKC, R.M. Epand outlines effects of PKC modulators on membrane properties, and M. Mosior and S. McLaughlin explain how electrostatics and dimensionality can produce apparent cooperativity when the PKC protein and its substrates bind to membranes. The latter chapter, even though informative, is probably the least directly relevant to scientists interested in PKC function. Most of these reviews provide useful and pertinent information. For example Epand highlights the surprising consequence of the fact that the activity of membrane-bound PKC can be regulated effectively by substances that do not bind to the enzyme with high affinity. This is because the volume of the membrane is small. Most PKC modulators are very lipophilic molecules which partition completely into the membrane phase and are thus concentrated in a small volume. For example, a modulator which is added to yield a concentration of 1 $\mu$M in an aqueous suspension of 10 $\mu g\, ml^{-1}$ membrane produces a concentration of 100 mM on complete partitioning into the membrane. This effect renders comparison of the potency of activators and inhibitors of PKC in different assays rather difficult.

Part 2 of the book comprises five chapters on studies of biological consequences of PKC function. H. Rasmussen, R.A. Calle, S. Ganesan, J.I. Smallwood, D.C. Throckmorton and W.S. Zawalich consider the role of PKC in sustained cellular responses, especially the spatial and temporal features of the events associated with sustained cellular responses. They also review the interaction between the signalling systems involving PKC and present a useful summary of the methods by which cellular PKC function can be studied. S. Jaken describes interactions of PKC isoenzymes with intracellular components. She points out that differences in subcellular location of PKC isoenzymes may provide a selective means for regulating accessibility to substrates which will not be apparent in *in vitro* assays. Immunocytofluorescence studies have shown that PKC isoenzymes are often linked to certain components of the cytoskeleton and the cell nucleus. The role of nuclear PKC is currently the topic of much debate. Increased expression of immediate early response genes occurs rapidly on PKC activation in many cell types, and it is conceivable that PKC in the nucleus is required for the regulation of nuclear events pivotal for growth and differentiation.

The function of PKC substrates is only poorly understood. The best known PKC substrate is a protein called MARCKS, which is an acronym for myristolylated alanine-rich C kinase substrate. In a chapter on 'the MARCKS protein: a PKC substrate which regulates cytoskeletal membrane interactions' by A. Aderem and J.T. Seykora the properties of this ubiquitous protein are introduced, and evidence is discussed which suggests that it cycles reversibly between the plasma membrane and the cytosol in a phosphorylation-dependent manner. Actin seems to be the cytoskeletal component of particular importance for the phosphorylation-sensitive binding of the MARCKS protein.

Second messenger pathways linked to PKC influence the properties of ion channels and thus the firing patterns of neurons. These pathways are reviewed by R.J. Knox and L.K. Kaczmarek. Probably the most interesting chapter of the book for the readers of this journal is the last one by C. Borner and D. Fabbro on PKC and cellular transformation. They integrate many of the findings outlined in the preceeding chapters in an attempt to conceptualise the role of PKC subtypes in the regulation of cell proliferation and intracellular communication. The action of PKC has to be seen as part of a growth factor-stimulated cascade of protein kinases which converges ultimately on the activity of a nuclear serine protein kinase called $p34^{cdc2}$. This enzyme regulates the transition of cells through mitosis by phosphorylation of lamins which are involved in the assembly of nuclear envelope proteins. The authors highlight recent biochemical and genetic approaches aimed at identifying mechanisms by which individual PKC subtypes contribute to cell transformation and tumour progression. They make the important points that an understanding of the role of PKC in normal cell growth requires the consideration of the cellular signalling as a whole and not of its parts in isolation, and that PKC has to be viewed not only as a positive growth effector but also as a negative feedback regulator which prevents overstimulation of cells and hence uncontrolled proliferation. These dual properties endow the PKC enzyme family with a multifunctional physiological role which is often ignored when PKC is claimed to be able to act as a transforming oncogene.

Overall the chapters are written in a fashion easily understood by biologists, pharmacologists and biochemists and by students in these disciplines. Each chapter is comprehensively referenced. The editors include also a very helpful glossary at the end of the book. If the book has weaknesses, they are those inevitably associated with multi-author collections of reviews on highly topical scientific issues: (i) there is considerable overlap between the different contributions, and (ii) findings published after 1991 are not included, which will make the book rapidly outdated. The urge for speedy publication is probably responsible for an unusually large number of trivial spelling mistakes. However these minor objections are easily outweighed by the usefulness of the overview provided. Nevertheless one has to agree with the conclusion drawn in chapter 10. 'In spite of a decade of intense effort since the discovery of PKC our knowledge of the cell biology and cellular functions of this ubiquitous family of enzymes is rudimentary.'

A. Gescher